# Fast Characterization of Inducible Regions of Atrial Fibrillation Models With Multi-Fidelity Gaussian Process Classification

**DOI:** 10.3389/fphys.2022.757159

**Published:** 2022-03-07

**Authors:** Lia Gander, Simone Pezzuto, Ali Gharaviri, Rolf Krause, Paris Perdikaris, Francisco Sahli Costabal

**Affiliations:** ^1^Center for Computational Medicine in Cardiology, Euler Institute, Università della Svizzera italiana, Lugano, Switzerland; ^2^Department of Mechanical Engineering and Applied Mechanics, University of Pennsylvania, Philadelphia, PA, United States; ^3^Department of Mechanical and Metallurgical Engineering, School of Engineering, Pontificia Universidad Católica de Chile, Santiago, Chile; ^4^Institute for Biological and Medical Engineering, Schools of Engineering, Medicine and Biological Sciences, Pontificia Universidad Católica de Chile, Santiago, Chile; ^5^Millennium Nucleus for Cardiovascular Magnetic Resonance, Santiago, Chile

**Keywords:** machine learning, cardiac electrophysiology, atrial fibrillation, Gaussian processes, Riemannian manifolds, active learning

## Abstract

Computational models of atrial fibrillation have successfully been used to predict optimal ablation sites. A critical step to assess the effect of an ablation pattern is to pace the model from different, potentially random, locations to determine whether arrhythmias can be induced in the atria. In this work, we propose to use multi-fidelity Gaussian process classification on Riemannian manifolds to efficiently determine the regions in the atria where arrhythmias are inducible. We build a probabilistic classifier that operates directly on the atrial surface. We take advantage of lower resolution models to explore the atrial surface and combine seamlessly with high-resolution models to identify regions of inducibility. We test our methodology in 9 different cases, with different levels of fibrosis and ablation treatments, totalling 1,800 high resolution and 900 low resolution simulations of atrial fibrillation. When trained with 40 samples, our multi-fidelity classifier that combines low and high resolution models, shows a balanced accuracy that is, on average, 5.7% higher than a nearest neighbor classifier. We hope that this new technique will allow faster and more precise clinical applications of computational models for atrial fibrillation. All data and code accompanying this manuscript will be made publicly available at: https://github.com/fsahli/AtrialMFclass.

## 1. Introduction

Atrial Fibrillation (AF) is the most common cardiac arrhythmia and a significant contributor to morbidity and mortality (Virani et al., 2021). AF is characterized by a chaotic electrical activity of the atria and perpetuated by multiple re-entrant wavelets propagating in the atrial tissue. It has been shown in several studies that in patients in the early stages of AF (paroxysmal AF), the chaotic activity is originated mainly from the pulmonary veins (PVs) (Haissaguerre et al., 1998; Chen et al., 1999). Thus, PV isolation (PVI) is the cornerstone of AF treatment at this point (Kawai et al., 2019). Here, ablation lines around the PVs are created to electrically isolate them. However, in patients with a persistent form of AF, PVI efficacy remains sub-optimal (Verma et al., 2015; Kawai et al., 2019). The detriment in the effect of this treatment in persistent AF patients is caused mainly by the shift of electrical abnormalities in the PVs to other locations and higher degrees of structural remodelling (Boyle et al., 2019; Kawai et al., 2019). Targeting arrhythmic substrates in persistent AF patients, in addition to PVI, could not demonstrate any benefit, as these treatment approaches do not incorporate strategies to find optimal ablation targets according to the AF mechanism (Verma et al., 2015). Furthermore, the high inter-individual variability in fibrosis distributions (McDowell et al., 2015; Boyle et al., 2019) and sources maintaining AF indicates an urgent need for patient-specific approaches.

Simulations, conducted in computational atrial models, have recently been used to develop mechanistic insights into the perpetuation and ablation of persistent AF patients with atrial fibrosis (McDowell et al., 2015; Boyle et al., 2019; Loewe et al., 2019; Roney et al., 2019). A common approach to investigate AF is to stimulate a high fidelity model from different pacing sites and observe whether this arrhythmia was induced or not (Boyle et al., 2019). With these simulations, it is possible to create an inducibility map that shows the regions of the atria where AF will manifest if stimulated (Potse et al., 2018). Moreover, this map can be reduced into one metric, the inducibility, which corresponds to the fraction of the tissue where AF can be induced. This quantity is useful to compare different ablation treatments, as the most efficient intervention will be the one that reaches the lowest inducibility with the lowest amount of ablation (Gharaviri et al., 2021a,b).

Inducibility maps are computationally expensive to compute with high fidelity models. The complete exploration of all the potential sites that could trigger an arrhythmia is currently unfeasible (Loewe et al., 2019). For this reason, a number of alternatives have been proposed. A viable option is to design a pacing protocol that maximizes the chance of inducing AF (Azzolin et al., 2021). Alternatively, the computational cost per simulation could be reduced by a faster implementation of the AF model, e.g., based on GPGPU (Kaboudian et al., 2019). Additionally, low fidelity models provide an approximation that could be based on simplified physics, e.g., eikonal models (Fu et al., 2013; Quaglino et al., 2018), reduced-order modeling (Fresca et al., 2020; Pagani and Manzoni, 2021) or simply on a coarser discretization (Quaglino et al., 2019; Dhamala et al., 2020).

Low fidelity models alone are faster, but potentially imprecise in reproducing the high fidelity inducibility map. However, a certain degree of statistical correlation between high- and low fidelity maps is to be expected. Multi-fidelity approaches can exploit this inter-model correlation to improve the accuracy of the estimators for a fixed total cost or, equivalently, to reduce the total cost of estimation for a targeted accuracy (Perdikaris et al., 2016; Quaglino et al., 2018, 2019; Sahli Costabal et al., 2019). This is achieved by offsetting most of the computational burden to the low fidelity model. Moreover, the overall computational cost could also be further reduced by carefully selecting the training points. To this end, Bayesian decision making strategies, commonly referred to as active learning (Cohn et al., 1996), can provide a principled way for judiciously selecting new observations towards improving classification accuracy. The process consists in adding points iteratively in the locations where the uncertainty is greater (Kapoor et al., 2007; Gramacy and Polson, 2017; Sahli Costabal et al., 2020; Zaman et al., 2021).

The problem of creating an inducibility map can be seen as a classification problem, from a machine learning perspective. The labels, in this case, are the occurrence or absence of AF when we pace the model from a specific site, which corresponds to the input. Although this may seem a trivial task, for which many tools are available, it is not straightforward when the classification domain is a Riemannian manifold, such as the atrial surface. In this case, points that may be close in the Euclidean space might be apart in the manifold due to its topology. There has been recent attention in the machine learning community on formulating effective Gaussian process (GP) models for supervised learning on Riemannian manifolds (Coveney et al., 2019; Borovitskiy et al., 2020). GPs tend to perform well when the amount of data available is limited, and, due to their Bayesian nature, they provide built-in uncertainty in the predictions. However, current approaches, also adopted in the cardiac modeling community, have focused on the regression case (Coveney et al., 2019; Coveney S. et al., 2020). Performing classification with Gaussian processes is a challenging task, as there is no closed expression of likelihood and requires different types of approximations to perform statistical inference (Rasmussen and Williams, 2006).

**Figure 1:**
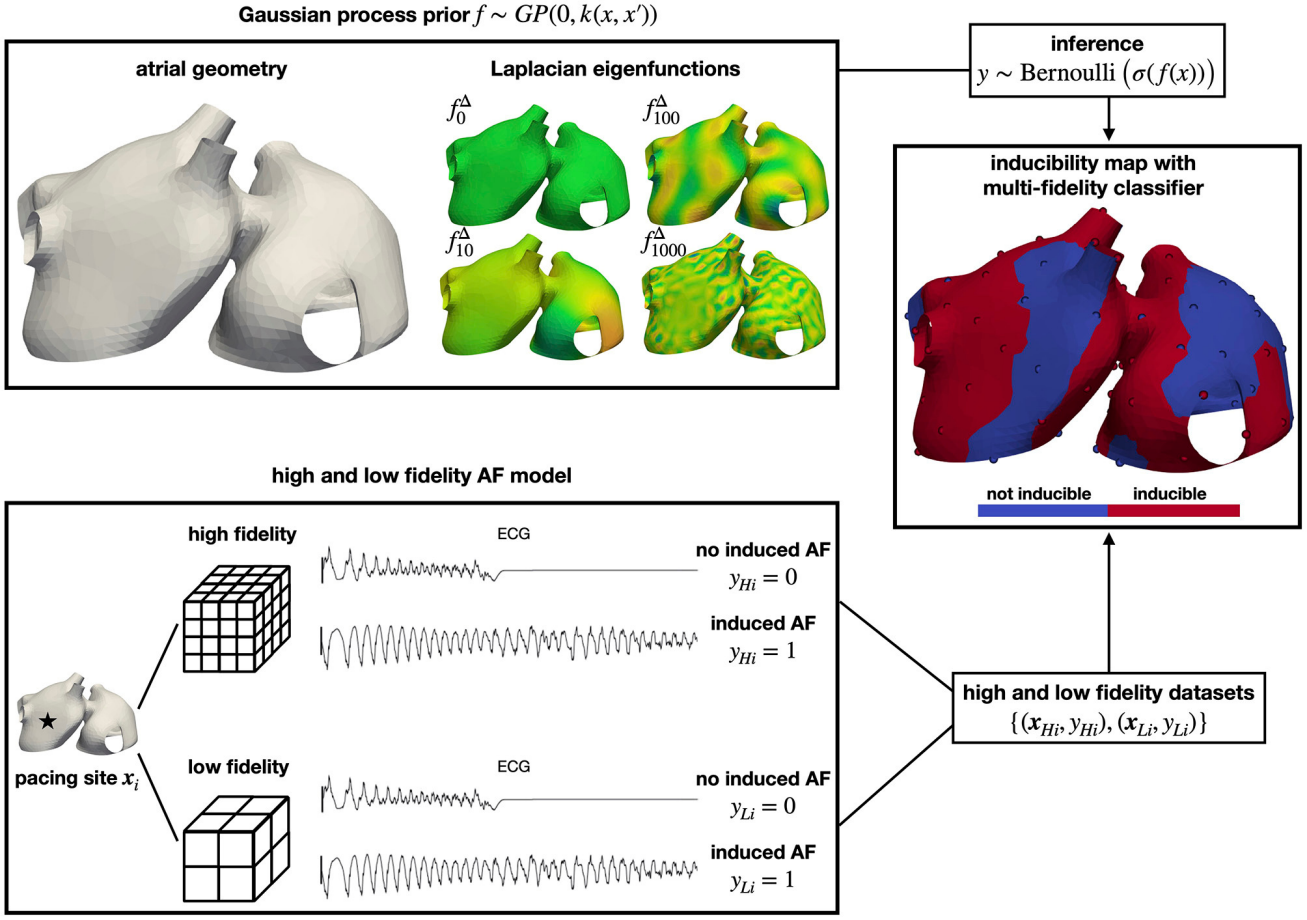
Overview of the methodology. We predict the regions where AF can be induced using a multi-fidelity Gaussian process classifier. We use the Laplacian eigenfunctions of the atrial geometry to efficiently construct a Gaussian process covariance function that operates directly on the manifold surface. We pace different sites in computational models of AF with low and high resolution to create a dataset to train our classifier. In the end, we obtain an inducibility map that can be used to assess treatments.

In this work, we develop GP classifiers that can operate on manifolds, such as the atrial surface (see Figure 1). We extend this tool to seamlessly combine different levels of data fidelity by creating a multi-fidelity GP classifier. In the specific context of AF, we aim to develop a method that allows us to comprehensively determine atrial regions, for a specific structural remodeling pattern, that, if stimulated could successfully initiate AF, creating an inducibility map *in-silico*. In particular, our low fidelity model is based on a coarser spatial discretization of the atrial geometry and on a larger time step in the solution of the electrophysiology equations. The inducibility map is reconstructed using a multi-fidelity GP classifier, resulting in a function on the atrial surface taking boolean values, depending on whether AF is or is not inducible when pacing from a given location. We will demonstrate that this approach is more efficient and accurate than other classifiers, and even single-fidelity methods, for cases with and without ablation treatments and for different fibrosis patterns.

The manuscript is organized as follows. In Section 2 we present the AF model and the classification method, obtained by extending the classic GP classification on manifolds. We also present the multi-fidelity approach, as well as the active learning scheme employed to sequentially acquire new information. Section 3 is devoted to the numerical experiments. Specifically, we propose a numerical assessment of the classifiers, including nine case studies involving the characterization of inducibility regions of atrial models. The discussion in Section 4 concludes the manuscript.

## 2. Methods

### 2.1. Atrial Modeling

In this work, we use previously developed highly detailed human atrial model of atrial fibrillation (AF) (Potse et al., 2018; Gharaviri et al., 2020). We briefly summarize here the relevant aspects of the model. The anatomy, including heart and torso, is based on MRI data. Several key features (bundles, fibers) are based on histological studies and added manually. The atrial wall is 3-dimensional with variable thickness.

**Figure 2:**
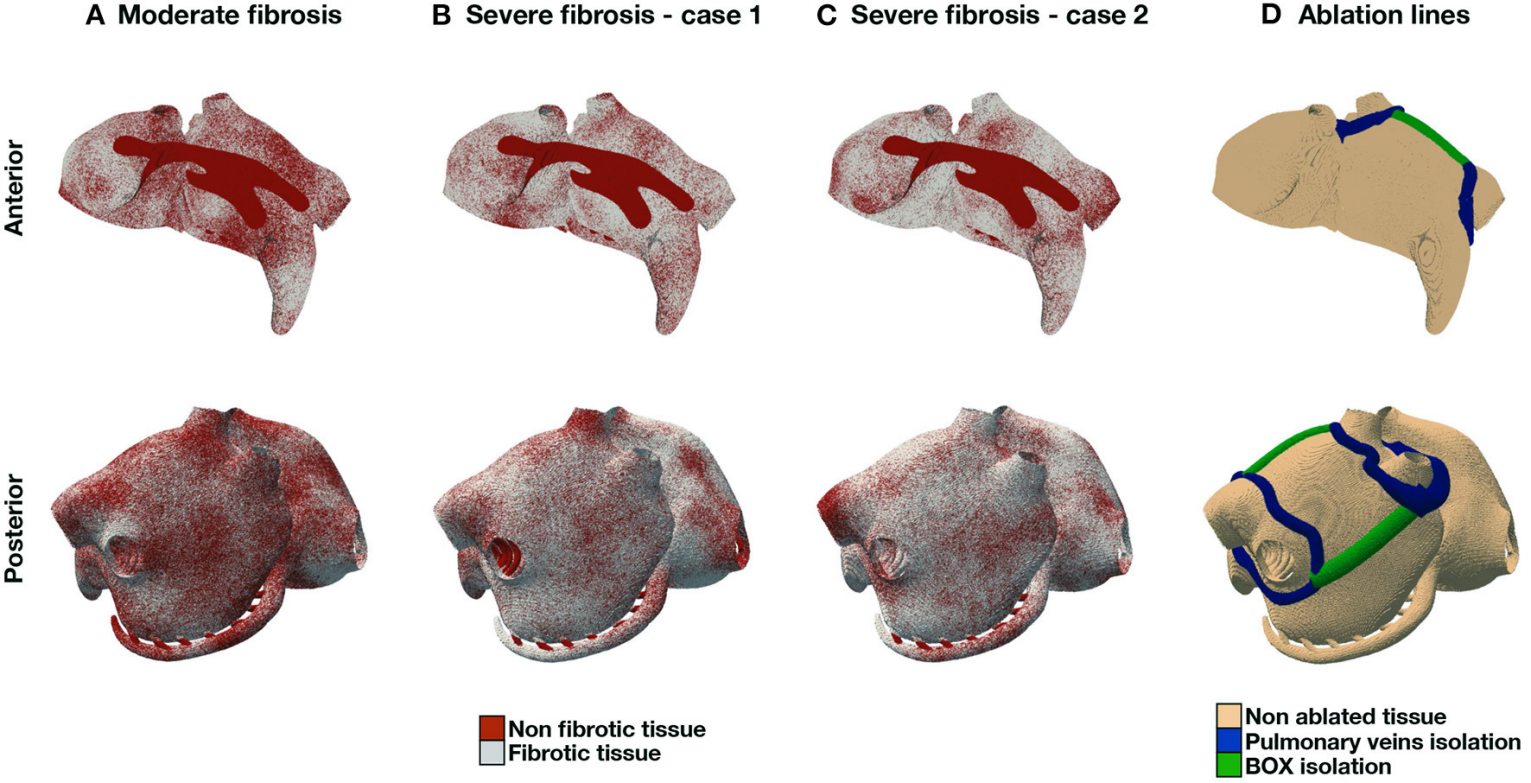
AF models. Fibrosis distribution in 3 different scenarios: moderate fibrosis [**(A)**, 50% fibrotic tissue], and severe fibrosis (70%) in two different patterns **(B), (C)**. **(D)** shows PVI and BOX ablation lines.

In the numerical experiments for this study, we consider different combinations of fibrosis patterns and ablation lines, for a total of 9 scenarios. Firstly, we consider three fibrosis patterns (Figure 2), one case with moderate fibrosis, corresponding to 50% of fibrotic tissue, and two cases with severe fibrosis, corresponding to 70% of fibrotic tissue. We consider endomysial fibrosis, which is modeled by formally imposing zero cross-fiber intracellular conductivity in fibrotic regions. Secondly, we implement two standard-of-care ablation strategies, pulmonary veins isolation (PVI) and PVI with roof lines (PVI+BOX), see Figure 2D. Ablation lines are non-conductive tissue.

The electrical activity is modeled with the monodomain system (Colli Franzone et al., 2014), which reads as follows

(1) $$\begin{array}{l} \{\begin{array}{ll} \chi (C_{\text{m}}\partial_{t}V_{\text{m}}+I_{\text{ion}}\left(V_{\text{m}},\text{w}\right)\\ \;+I_{\text{stim}}\left(\text{x},t\right))=\nabla \cdot \left({\text{G}}_{\text{m}}\nabla V_{\text{m}}\right), & \left(\text{x},t\right)\in \Omega \times \left(0,T\right],\\ \partial_{t}\text{w}=\text{g}\left(V_{\text{m}},\text{w}\right), & \left(\text{x},t\right)\in \Omega \times \left(0,T\right],\\ {\text{G}}_{\text{m}}\nabla V_{\text{m}}\cdot \text{n}=0, & \left(\text{x},t\right)\in \partial \Omega \times \left(0,T\right],\\ V_{\text{m}}\left(\text{x},0\right)=V_{0},\;\text{w}\left(\text{x},0\right)={\text{w}}_{0}, & \text{x}\in \Omega , \end{array} \end{array}$$

where *V*_m_(**x**, *t*) is the transmembrane potential, **w**(**x**, *t*) is a vector of ion gating and concentration variables, Ω is a domain describing the active myocardium, *C*_m_ = 1 μF cm^−2^ is the membrane capacitance, χ = 800 cm^−1^ is the membrane surface-to-volume ratio, *I*_stim_ is the current stimulus, **G**_m_(**x**) is the monodomain conductivity tensor, and *I*_ion_ and **g** describe the ionic model. In particular, we consider the Courtermanche-Ramirez-Nattel model (Courtemanche et al., 1998) adapted to an AF phenotype, with minor adaptations to guarantee numerical stability when evaluating the gating parameters for certain values of *V*_m_ (Potse, 2019). The initial condition (*V*_0_, **w**_0_) corresponds to the resting state.

The conductivity tensor **G**_m_ is defined as ${\text{G}}_{\text{i}}{\left({\text{G}}_{\text{i}}+{\text{G}}_{\text{e}}\right)}^{-1}{\text{G}}_{\text{e}}$, where **G**_i_ and **G**_e_ are, respectively, the intra- and extra-cellular conductivity tensors, both assumed transversely isotropic with respect to the local fiber direction. The intracellular longitudinal and cross conductivity are, respectively, set 3 and 0.3 mS cm^−1^, while the extracellular conductivities are 3 and 1.2 mS cm^−1^, respectively. The resulting conduction velocity in the fiber direction is 55.6 cm s^−1^. In the Bachmann's bundle, faster conduction is obtained with a longitudinal intracellular conductivity of 9 mS cm^−1^. Finally, the region between the superior and inferior vena cava is assumed isotropic, with all conductivities set to 1.5 mS cm^−1^.

The numerical solution of Equation (1) is based on a second-order finite difference scheme for the spatial discretization, and a fully explicit first-order Euler scheme for time stepping (Potse et al., 2006). The Rush-Larsen scheme is adopted to update the gating variables. The computational domain is discretized using a uniform mesh with hexahedral elements of side *h*.

For the high fidelity simulations, we consider a fine mesh with *h* = 0.2 mm and a time step of Δ*t* = 0.01 ms. For the low fidelity simulations, we double the discretization parameters, with *h* = 0.4 mm and Δ*t* = 0.02 ms. The coarsening of the grid is performed by employing a majority rule to determine the tissue type and fiber orientation of the coarse hexahedral elements from the eight sub-elements of the fine mesh. Moreover, the coarse model assumes a reduced surface-to-volume ratio χ = 450 cm^−1^ to balance out the expected reduction in conduction velocity due to a coarser space discretization (Pezzuto et al., 2016).

All simulations are performed with the Propag-5 software (Potse et al., 2006; Krause et al., 2012) on the Swiss National Supercomputing Centre (CSCS). For one simulation with *T* = 4 s, the compute time of the high fidelity model is 1 h40 min with 8 nodes, whereas the compute time of the low fidelity model is 14 min with 4 nodes. This means that the low fidelity model is approximately 16 times faster than the high fidelity model.

### 2.2. Pacing Protocol for Atrial Fibrillation

**Figure 3:**
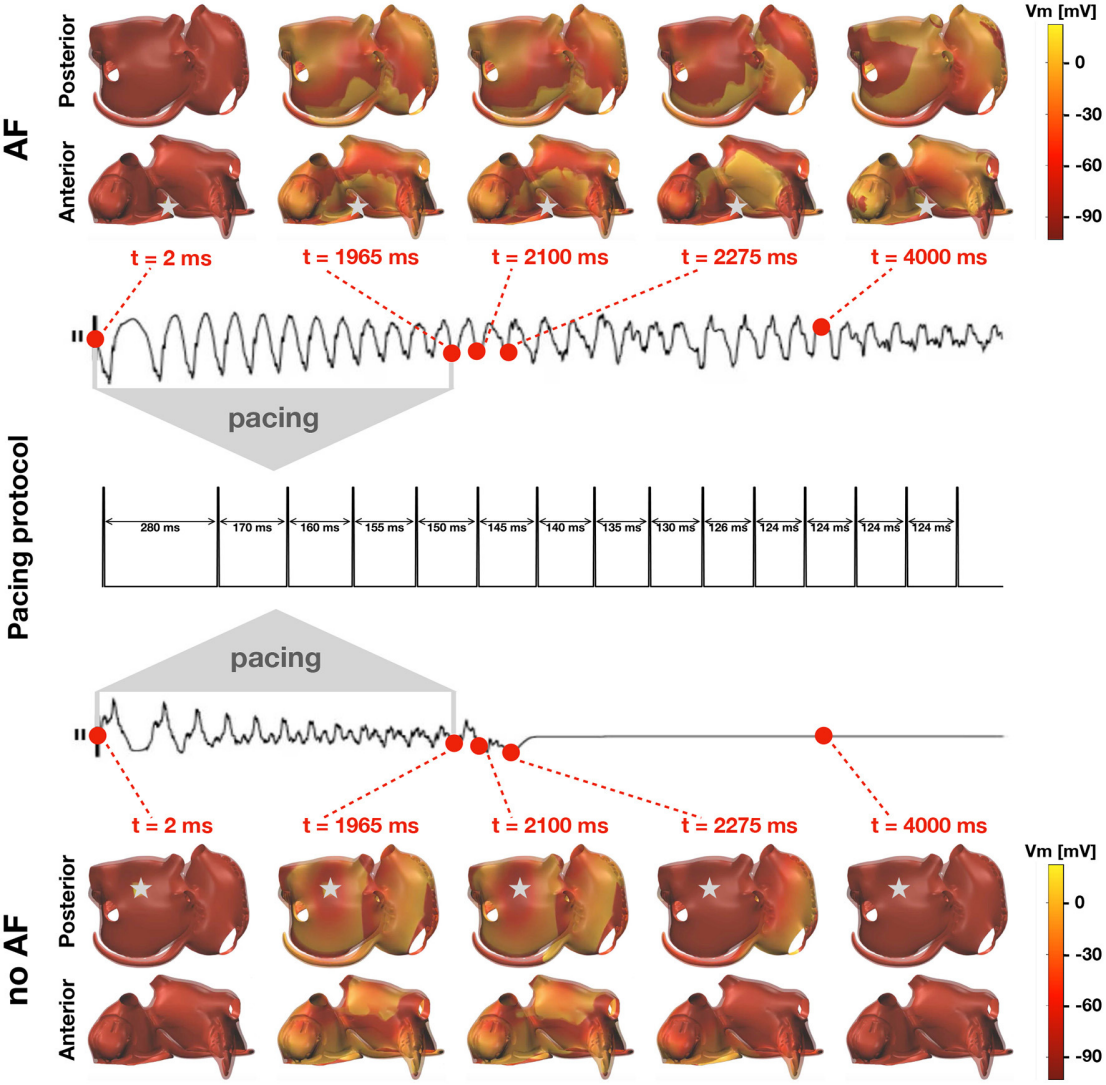
Inducibility of AF in the computer model. Two simulations with different pacing sites (grey stars) and inducibility outputs. The middle plot illustrates the pacing protocol. Top: transmembrane potential resulting from a successful induction of AF and corresponding lead II ECG recording. The stimulation results in a self-sustained activity. Bottom: transmembrane potential resulting from an unsuccessful induction of AF and corresponding lead II ECG recording. The stimulation results in a vanishing wave.

The stimulation protocol, encoded in the function *I*_stim_(**x**, *t*), is defined by a point **x**_stim_ ∈ Ω and a vector of distinct times $\tau_{\text{stim}}={\left\{\tau_{j}\right\}}_{j=1}^{N_{\text{stim}}}$ through the expression

(2) $$\begin{array}{l} I_{\text{stim}}\left(\text{x},t;{\text{x}}_{\text{stim}},\tau_{\text{stim}}\right)=\\ \{\begin{array}{ll} I_{\text{max}}, & \left(\text{x},t\right)\in B_{r}\left({\text{x}}_{\text{stim}}\right)\times {⋃}_{j=1}^{N_{\text{stim}}}\left[\tau_{j},\tau_{j}+\Delta \tau \right],\\ 0, & \text{otherwise}, \end{array} \end{array}$$

where *B*_*r*_(**x**_stim_) = {**x** ∈ Ω:**x**_stim_ ≤ **x** ≤ **x**_stim_ + *r*} is a *r*-neighborhood (the ≤ is meant component-wise) of the stimulation site, and Δτ > 0 is the stimulus duration. In this study, the vector ***τ***_stim_ is fixed as in Figure 3 (middle panel), which consists in a series of *N*_stim_ = 14 stimuli with decreasing temporal distance, whereas **x**_stim_ varies for each simulation. Each stimulus lasts Δτ = 4 ms, and has a strength *I*_max_ = 800 μA cm^−2^ with a fixed radius *r* = 0.8 cm, which is enough to maximize the chance that the tissue correctly captures it (Potse et al., 2018). The induction of AF is not successful when (*V*_m_, **w**) asymptotically approaches the resting state after the delivery of the last stimulus. Otherwise, if a self-sustained activity is still present at the end of the simulation, the induction of AF is successful. The idea is summarized in Figure 3. For sake of simplicity, in this work there is no distinction between a true AF episode and an atrial flutter, which could be understood as a periodic solution of the monodomain system.

The objective of this work is to learn the set A⊂Ω, such that if ${\text{x}}_{\text{stim}}\in A$ a sustained episode of AF is observed. In particular, we are interested in approximating the indicator function of A, denoted by **F**:Ω → {0, 1} such that ${\text{F}}^{-1}\left(1\right)=A$. The overall inducibility, which reflects the fraction of the tissue where AF can be initiated, follows immediately from the definition of A as

$$I=\frac{\left|A\right|}{\left|\Omega \right|}=\frac{1}{\left|\Omega \right|}\int_{\Omega }F\left(\text{x}\right)\text{ d}\text{x}.$$

Interestingly, the formula generalizes to the case of non-uniformly distributed ectopic foci. Let ρ(**x**) be the probability density function of the distribution of foci, then the inducibility can be obtained as

$$I_{\rho }=\int_{\Omega }F\left(\text{x}\right)\rho \left(\text{x}\right)\text{ d}\text{x}.$$

In this way, for instance, it is possible to account for a higher density of ectopic activity around the pulmonary veins and fibrotic regions. In this work, we will only consider a uniform distribution of foci, equivalent to select ρ(**x**) = |Ω|^−1^.

### 2.3. Classification With Gaussian Processes

Next, we present the proposed methodology for learning the inducibility function **F** from a limited set of simulations. We start by assuming that we have a data-set of *N* input/output pairs $D={\left\{\left({\text{x}}_{i},y_{i}\right)\right\}}_{i=1}^{N}$, where **x**_*i*_ ∈ Ω and *y*_*i*_ ∈ {0, 1}. Since the atrial wall is thin, we constrain the points to belong to a mid-wall smooth atrial surface S⊂Ω. We remark however that there is no loss of generality in the following presentation, as the methodology applies to the volumetric domain Ω in the same manner. Moreover, since *y*_*i*_ takes only binary values, we also restrict the scope of this work to binary classification. We also note that it is straightforward to extend this framework to the multi-class classification setting.

The classical formulation of Gaussian process classification defines an inter mediate variable which is computed from a latent function *f*(**x**) (Rasmussen and Williams, 2006). Throughout this article, we will assume standardized data-sets and work with zero-mean Gaussian process priors of the form $f\sim GP\left(0,k\left(\text{x},{\text{x}}^{\prime };\theta \right)\right)$. Here, *k*(·, ·; θ) is a covariance kernel function, which depends on a set of parameters θ. We adopt a fully Bayesian treatment and prescribe prior distributions over these parameters, which we will specify later (Neal, 1999). To obtain class probability predictions we pass the Gaussian process output *f* through a non-linear warping function σ:ℝ → [0, 1], such that the output is constrained to [0, 1], rendering meaningful class probabilities. We define the conditional class probability as π(**x**) = ℙ[*y* = 1|**x**] = σ(*f*(**x**)). A common choice for σ(*f*) is the logistic sigmoid function σ(*f*) = (1 + exp(−*f*))^−1^, which we will use throughout this work. We assume that the class labels are distributed according to a Bernoulli likelihood with probability σ(*y*) (Nickisch and Rasmussen, 2008).

### 2.4. Gaussian Process Priors on Manifolds

A crucial step in building a Gaussian process classifier is the choice of the kernel function. A popular choice is the Matérn kernel, which explicitly allows one to encode smoothness assumptions for the latent functions *f*(**x**) (Rasmussen and Williams, 2006). In a Euclidean space setting, the kernel function has the form (Rasmussen and Williams, 2006):

(3) $$\begin{array}{l} k\left(\text{x},{\text{x}}^{\prime },\theta \right)=\eta^{2}\frac{2^{1-\nu }}{\Gamma \left(\nu \right)}{\left(\sqrt{2\nu }\frac{\left||\text{x}-{\text{x}}^{\prime }|\right|}{\ell }\right)}^{\nu }K_{\nu }\left(\sqrt{2\nu }\frac{\left||\text{x}-{\text{x}}^{\prime }|\right|}{\ell }\right), \end{array}$$

where Γ is the gamma function, and *K*_ν_ is the modified Bessel function of the second kind. The parameter η controls the overall variance of the Gaussian process, the parameter ℓ controls the spatial correlation length-scale, and ν controls the regularity of the latent functions *f*(**x**) (Rasmussen and Williams, 2006). When ν → ∞, we recover the popular squared exponential kernel, also known as radial basis function, that yields a prior over smooth functions with infinitely many continuous derivatives.

The form presented in Equation (3) is not suitable to be used on manifolds, as the atrial surface. A naive approach is to replace the Euclidean distance between points with the geodesic distance on the manifold surface. Even though this approach may work for some cases, there is no guarantee that the resulting covariance matrix between input points will be positive semi-definite (Pezzuto et al., 2019; Borovitskiy et al., 2020), a key requirement for a kernel function. As a matter of fact, the choice of the kernel is problematic in this case. For instance, the Matérn family does not yield positive definite kernels even on the sphere, except for a few exceptional choices of the parameters (Gneiting, 2013). Here, we follow an alternative approach, implicitly based on the solution of the following stochastic partial differential equation (SPDE) (Whittle, 1963; Lindgren et al., 2011):

(4) $$\begin{array}{l} \{\begin{array}{ll} {\left(\kappa^{2}\text{I}-\Delta \right)}^{\nu /2+d/4}u=W, & \text{x}\in \Omega ,\\ \text{n}\cdot \nabla {\left(\kappa^{2}\text{I}-\Delta \right)}^{j}u=0, & \text{x}\in \partial \Omega \text{,}j=0,\ldots ,\left\lfloor \frac{\nu -1}{2}+\frac{d}{4}\right\rfloor , \end{array} \end{array}$$

where −Δ is the Laplace-Beltrami operator on the *d*-dimensional manifold, and W is the spatial Gaussian white noise on Ω. When Ω = ℝ^*d*^, the solution of the fractional SPDE is a Matérn random field with $\kappa =\frac{\sqrt{2\nu }}{\ell }$ (Lindgren et al., 2011). However, compared to Equation (3), the SPDE in Equation (4) trivially generalizes to manifolds with no loss of positive definiteness of the correlation kernel, thanks to the properties of the pseudo-differential operator (Borovitskiy et al., 2020). The correlation function can be explicitly written as follows. Let ${\left\{\left(\lambda_{i},\psi_{i}\right)\right\}}_{i=0}^{\infty }$ be the eigenvalue/eigenfunction pairs of the Laplace-Beltrami operator with pure Neumann boundary conditions, that is

(5) $$\begin{array}{l} \{\begin{array}{ll} -\Delta \psi_{i}=\lambda_{i}\psi_{i} & \text{x}\in \Omega ,\\ -\text{n}\cdot \nabla \psi_{i}=0, & \text{x}\in \partial \Omega , \end{array} \end{array}$$

for all *i* ∈ ℕ. Then, we can represent Matérn-like kernels on manifolds as (Coveney et al., 2019; Borovitskiy et al., 2020).

(6) $$\begin{array}{l} k\left(\text{x},{\text{x}}^{\prime };\theta \right)=\frac{\eta^{2}}{C}\overset{\infty }{\underset{\text{i}=0}{\sum }}{\left(\frac{1}{\ell^{2}}+\lambda_{i}\right)}^{-\nu -\frac{d}{2}}\psi_{i}\left(\text{x}\right)\psi_{i}\left({\text{x}}^{\prime }\right) \end{array}$$

where *C* is a normalizing constant. This eigen-decomposition also enables a direct solution of the SPDE, providing the following representation of the Gaussian process prior:

(7) $$\begin{array}{l} f\left(\text{x}\right)\approx \frac{\eta^{2}}{C}\overset{\infty }{\underset{\text{i}=0}{\sum }}w_{i}{\left(\frac{1}{\ell^{2}}+\lambda_{i}\right)}^{-\frac{\nu }{2}-\frac{d}{4}}\psi_{i}\left(\text{x}\right),\;w_{i}\sim N\left(0,1\right) \end{array}$$

In practice, the eigen-decomposition is truncated to a number *N*_eig_ of pairs.

In this work, we discretize the manifold $S\subset {\mathbb{R}}^{3}$ using a triangulated mesh and solve Equation (5) using finite element shape functions. As such, we can obtain the stiffness matrix ***A*** and mass matrix ***M***:

(8) $$\begin{array}{ll} A_{ij}=\overset{n_{\text{el}}}{\underset{\text{e}=1}{\text{A}}}\int_{B}\nabla N_{j}\cdot \nabla N_{i}\;dx, & M_{ij}=\overset{n_{\text{el}}}{\underset{\text{e}=1}{\text{A}}}\int_{B}N_{j}N_{i}\;dx, \end{array}$$

where A represents the assembly of the local element matrices, and *N* are the finite element shape functions. Then, we solve the eigenvalue problem:

(9) $$\begin{array}{l} Av=\lambda Mv \end{array}$$

In practice, to compute the kernel in Equation (6) we use a portion of all the resulting eigenpairs, starting from the smallest eigenvalues. We also use the corresponding eigenvectors as the eigenfunctions with *f*(***x***_i_) = ***v***_i_, where i is the node index at location **x**_i_. Given that the eigenvalue problem is solved only once as a pre-processing step, this methodology provides an efficient way to compute the kernel and the prior in a manifold.

### 2.5. Bayesian Inference

We finalize our Bayesian model description by prescribing the prior distributions for the kernel parameters. We assume the following distributions for the parameters θ = {η, ℓ},

(10) $$\begin{array}{l} \eta \sim \text{HalfNormal}\left(\sigma =10000\right) \end{array}$$

(11) $$\begin{array}{l} \ell \sim \text{Gamma}\left(\alpha =1,\beta =1\right). \end{array}$$

The posterior distribution over the model parameters θ = {η, ℓ} cannot be described analytically, and thus we must resort to approximate inference techniques to calibrate this Bayesian model on the available data. To this end, we use the NO-U-Turn sampler (NUTS) (Hoffman and Gelman, 2014), which is a type of Hamiltonian Monte Carlo algorithm, as implemented in NumPyro (Phan et al., 2019). We use one chain, and set the target accept probability to 0.9. The first 500 samples are used to adjust the step size of the sampler, and are later discarded. We use the subsequent 500 samples to statistically estimate the parameters θ.

Once we have completed the inference, we can make predictions y^*^ at new locations x^*^ in three steps. First, we compute the predictive posterior distribution of the latent function $f^{*}\left(x^{*}\right)\sim N\left(\mu \left(x^{*}\right),\Sigma \left(x^{*}\right)\right)$, which by construction follows a multi-variate normal distribution, with a mean **μ** and covariance Σ obtained by conditioning on the available training data (Rasmussen and Williams, 2006):

(12) $$\begin{array}{l} \mu \left(x^{*}\right)=k\left(x^{*},X\right)K^{-1}f \end{array}$$

(13) $$\begin{array}{l} \Sigma \left(x^{*}\right)=k\left(x^{*},x^{*}\right)-k\left(x^{*},X\right)K^{-1}k\left(X,x^{*}\right), \end{array}$$

where the covariance matrix ***K*** ∈ ℝ^*N* × *N*^ results from evaluating the kernel function *k*(·, ·; θ) at the locations of the input training data ***X*** and ***f*** = *f*(***X***), respectively. We then proceed by sampling μ, Σ using model parameters drawn from the estimated posterior distributions of θ and ***f***. This will result in a number of random variables f^*^ that are independent and normally distributed, which we can be used to compute statistical averages as

(14) $$\begin{array}{l} {\hat{f}}^{*}\sim N\left(\hat{\mu },\hat{\Sigma }\right),\;\hat{\mu }=\frac{1}{N_{\text{s}}}\overset{N_{\text{s}}}{\underset{\text{i}=1}{\sum }}\mu_{i},\;\hat{\Sigma }=\frac{1}{N_{\text{s}}}\overset{N_{\text{s}}}{\underset{\text{i}=1}{\sum }}\Sigma_{i}, \end{array}$$

where *N*_s_ is the number of samples considered for θ and ***f***. We finally pass ${\hat{f}}^{*}$ through the logistic sigmoid function σ to obtain a distribution of class probabilities y^*^.

### 2.6. Multi-Fidelity Classification With Gaussian Processes

In this work, we will assume that we have 2 information sources of different fidelity. We will call the high fidelity, computationally expensive, and hard to acquire information source with the subscript *H* and the inexpensive, faster to compute, low fidelity source with the subscript *L*. Now, our data set comes from these two sources $D=\left\{{\left(x_{Li},y_{Li}\right)}_{i=1}^{N_{L}},{\left(x_{Hi},y_{Hi}\right)}_{i=1}^{N_{H}}\right\}=\left\{X,y\right\}$. We will postulate two latent functions *f*_*H*_ and *f*_*L*_, respectively, that are related through an auto-regressive prior (Kennedy and O'Hagan, 2000).

(15) $$\begin{array}{l} f_{H}\left(x\right)=\rho f_{L}\left(x\right)+\delta \left(x\right) \end{array}$$

Under this model structure, the high fidelity function is expressed as a combination of the low fidelity function scaled by ρ, corrected with another latent function δ(***x***) that explains the difference between the different levels of fidelity. Following (Kennedy and O'Hagan, 2000), we assume Gaussian process priors on these latent functions.

(16) $$\begin{array}{l} f_{L}\sim GP\left(0,k\left(x,x^{\prime };\theta_{L}\right)\right) \end{array}$$

(17) $$\begin{array}{l} \delta \sim GP\left(0,k\left(x,x^{\prime };\theta_{H}\right)\right). \end{array}$$

The vectors ***θ***_*L*_ and ***θ***_*H*_ contain the kernel hyper-parameters of this multi-fidelity Gaussian processes model. The choice of the auto-regressive model leads to a joint prior distribution over the latent functions that can be expressed as (Kennedy and O'Hagan, 2000).

(18) $$\begin{array}{l} f=\left[\begin{array}{c} f_{L}\\ f_{H} \end{array}\right]\sim N\left(\left[\begin{array}{c} 0\\ 0 \end{array}\right],\left[\begin{array}{ll} K_{LL} & K_{LH}\\ K_{LH} & K_{HH} \end{array}\right]\right), \end{array}$$

with

(19) $$\begin{array}{l} \begin{array}{c} K_{LL}=\;k_{L}\left(X_{L},X_{L}^{\prime };\theta_{L}\right)\\ K_{LH}=\rho k_{L}\left(X_{L},X_{H}^{\prime };\theta_{L}\right)\\ K_{HH}=\rho^{2}k_{L}\left(X_{H},X_{H}^{\prime };\theta_{L}\right)+k_{H}\left(X_{H},X_{H}^{\prime };\theta_{H}\right). \end{array} \end{array}$$

The global covariance matrix ***K*** of this multi-fidelity Gaussian process model has a block structure corresponding to the different levels of fidelity, where ***K***_*HH*_ and ***K***_*LL*_ model the spatial correlation of the data observed in each fidelity level, and ***K***_*LH*_ models the cross-correlation between the two levels of fidelity. We also have kernel parameters for the different levels of fidelity. We again use the Matérn as described in Section 2.4, which results in parameters θ_*H*_ = (η_*H*_, ℓ_*H*_), and θ_*L*_ = (η_*L*_, ℓ_*L*_). For these parameters and the scaling factor ρ, we consider the following prior distributions

(20) $$\begin{array}{l} \eta_{H},\eta_{L}\sim \text{HalfNormal}\left(\sigma =10000\right) \end{array}$$

(21) $$\begin{array}{l} \ell_{H},\ell_{L}\sim \text{Gamma}\left(\alpha =2,\beta =2\right) \end{array}$$

(22) $$\begin{array}{l} \rho \sim \text{Normal}\left(\mu =0,\sigma =10\right). \end{array}$$

We can perform inference and prediction for this model in the same way as for the single fidelity classifier, as detailed in Section 2.5. In particular, we can use Equations (12) and (13) with the entire covariance matrix ***K*** to obtain the conditional mean and covariance of f^*^.

### 2.7. Active Learning

Here, we take advantage of the uncertainty predictions that are inherent to Gaussian processes and are absent in other types of classifiers, such as nearest neighbor. Specifically, at each active learning iteration, we train the classifier, and select the next point that should be included in our training data-set by solving the following optimization problem (Kapoor et al., 2007; Sahli Costabal et al., 2019):

(23) $$\begin{array}{l} x^{new}=\underset{x\in X_{cand}}{arg min}\frac{\left|\hat{\mu }\left(x\right)\right|}{\sqrt{\hat{\Sigma }\left(x\right)}}, \end{array}$$

where ***X***_*cand*_ represents a set of candidate locations that can be acquired. In our case, we use all the nodes in the mesh as candidates, except the ones at the boundaries which have artificially high variance. This active learning criterion presents a good balance between exploitation (sampling near the classification decision boundary) and exploration (discovering new inducible regions). It can be seen as promoting the selection of points that tend to be located near the decision boundary (σ(μ = 0) = 0.5), or points in regions with high uncertainty (as reflected by the posterior variance Σ). We keep adding points via this sequential active learning procedure until we have reached the desired number of samples.

## 3. Numerical Experiments

### 3.1. Numerical Assessment

We first create a synthetic example to test the performance of the proposed classifier. We study the length scale of different random fields that could represent the potential inducibility maps that we want to approximate in this study. In particular, we use a mesh based on the anatomy of the mid-layer described in Section 2.1. Here, we represent the left and right atria with 3,298 nodes and 6,335 triangles. First, we normalize the geometry by the largest standard deviation of one of its coordinates. In this way, we can use the same prior distributions regardless the particular geometry. Then, we generate Gaussian random fields on the atrial manifold with zero mean and the Matérn covariance kernel, as detailed in Equation (6). We use 1,000 eigenpairs to construct a computable kernel function approximation with ν = 3/2 and η = 1. We consider different length scales to simulate inducibility regions and assess the performance of the classifier: ℓ = {0.2, 0.4, 0.6, 0.8, 1.0}. Finally, we pass the resulting random field through the sigmoid function σ to obtain values between zero and one, which we round to the nearest integer to create discrete labels.

**Figure 4:**
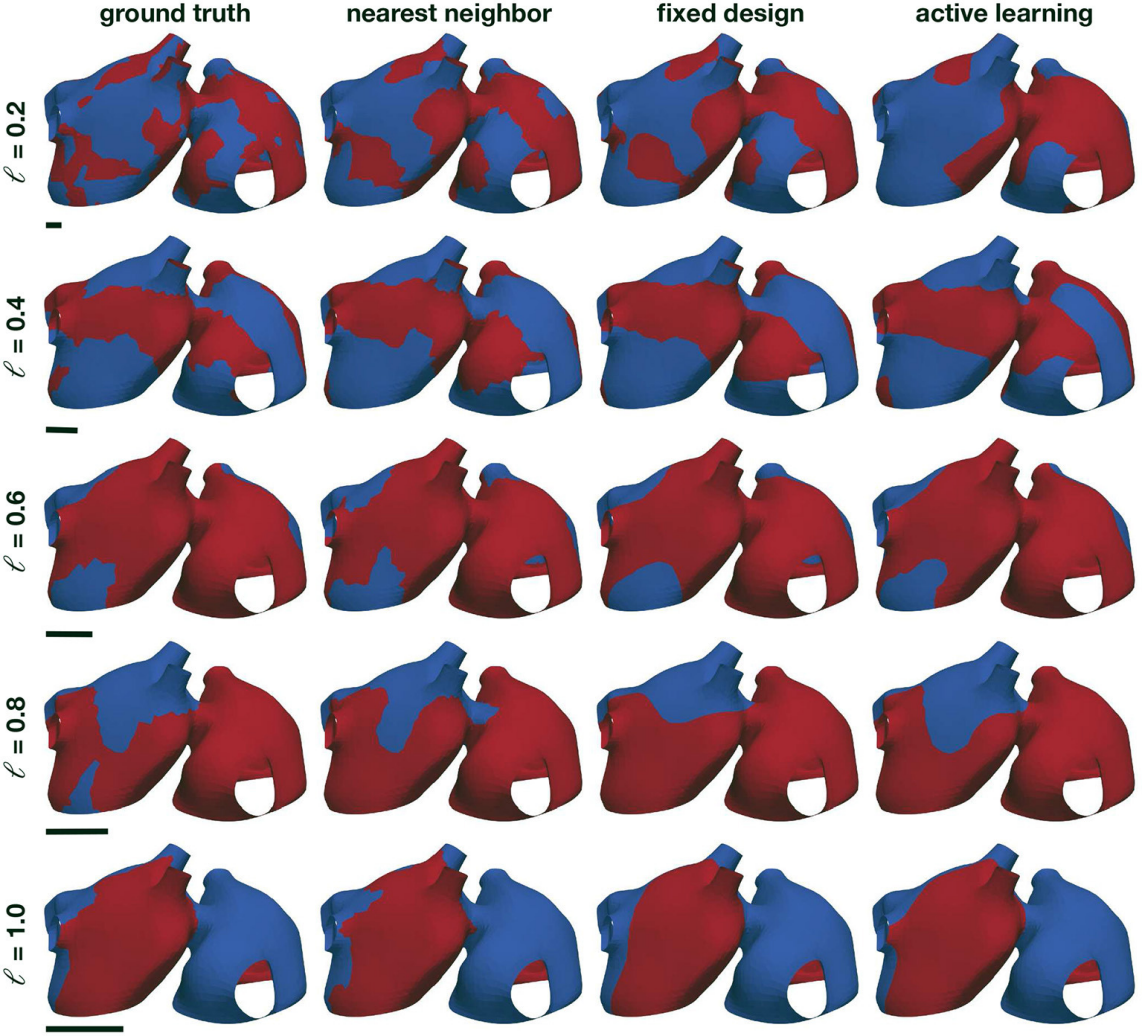
Numerical assessment of the Gaussian process classifier. We create different random examples with different correlations lengths (first column) and train a nearest neighbor classifier (second column), a Gaussian process classifier trained with the same data-set as the nearest neighbor classifier (third column), and Gaussian process classifier that adaptively selects the training points through active learning (fourth column). The black bars represent the size of the length scale relative to the atrial geometry.

Examples of the resulting random fields can be seen in Figure 4, left column. We compare three different classifiers. First, as a baseline benchmark, we create a nearest neighbor classifier. Here, the prediction of an unknown point is based on the label of the closest data point. Since we are working with a manifold, we use the geodesic distance to find the closest point, which we compute using the heat method (Crane et al., 2013). As a data-set, we use a fixed design spread through the manifold surface. To select the locations, we first randomly pick a node in the mesh, and then we add the node that is further away from the initial node using the geodesic distance. Then, we iterate, finding the point that is further away from all the nodes already included in the data-set, until we reached the desired data-set size. The second classifier that we consider is a Gaussian process classifier, as described in the previous sections, that is trained on the same fixed experimental design. The final classifier is also a Gaussian process classifier, which we train with the first 20 samples of the fixed experimental design, and then we apply the proposed active learning procedure. For all the Gaussian process classifiers in these experiments, we set the number of eigenfunctions used to *N*_eig_ = 1,000.

In these examples, we test the performance of the three different classifiers, using between 20 and 100 samples, and 10 different random fields for each of the 5 length scales selected. To take into account potential imbalances of classes in the examples generated, we use the balanced accuracy score. This metric is defined as the arithmetic mean of the sensitivity and specificity as

(24) $$\begin{array}{r} \text{balanced accuracy}=\\ \frac{1}{2}\left(\frac{\text{\# of predicted positives}}{\text{\# of real positives}}+\frac{\text{\# of predicted negatives}}{\text{\# of real negatives}}\right). \end{array}$$

In contrast to conventional accuracy, this metric will reflect if a classifier is predominately predicting one class due to the higher proportion of samples present in the data-set.

**Figure 5:**
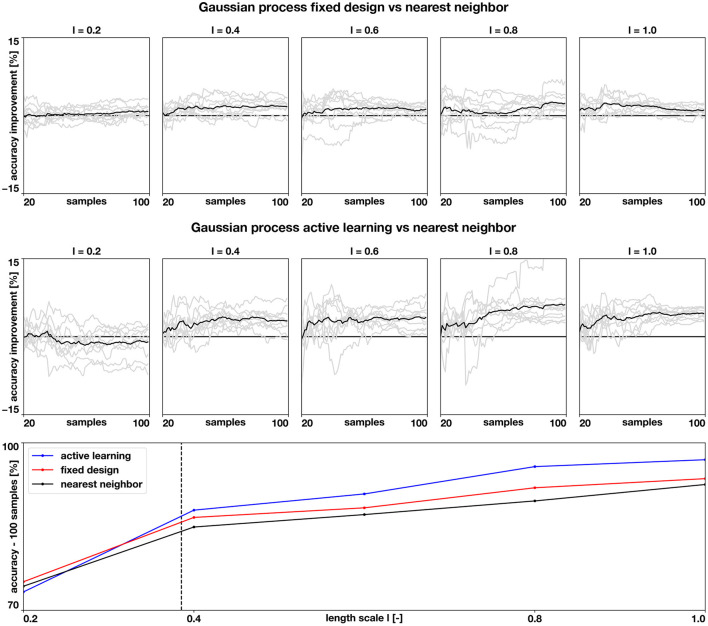
Accuracy of the numerical assessment. We quantify the improvements in accuracy when using a Gaussian process classifier versus the nearest neighbor classifier (top row) and when using a Gaussian process classifier with active learning, versus the baseline nearest neighbor classifier (middle row) for different length scales. The gray lines show the balanced accuracy improvements of the 10 examples for each length scale and the black line shows the mean improvement. The bottom row shows how the average balanced accuracy changes with length scale when the classifiers are trained with 100 samples. The dashed vertical line represents the average geodesic distance between training points of the fixed design.

The results of this assessment are summarized in Figures 4, 5. We first observe in Figure 4, left column, that the complexity of the classification regions increases as the correlation length scale is reduced. In the same figure, we show the different classifiers trained with 100 samples. It is visually possible to note that the accuracy of the classifiers degrades as the length scale of the ground-truth classification surface is decreased. For the length scale ℓ = 0.2, some regions are not captured by the classifiers. We also note that the Gaussian process classification boundaries tend to be smoother than the nearest neighbor classifier. These differences are quantified in Figure 5. We first compare the improvements in accuracy between the nearest neighbor classifier and the Gaussian process classifier with a fixed design in the top row. These two methods are trained with identical data, and we observe that for most cases and number of samples, the Gaussian process classifier is more accurate than the nearest neighbor classifier. The accuracy improvements at 100 samples range on average from 0.8% at ℓ = 0.2 to 2.4% at ℓ = 0.8. Then, we compare the nearest neighbor classifier with the Gaussian process classier trained with active learning. These two classifiers only share the first 20 points of data. Then the active learning classifier judiciously selects the remaining samples attempting to maximize accuracy. We observe that the accuracy improvements are more pronounced with the active learning for ℓ = 0.4 − 1.0. The average improvements at 100 samples range from 3.0% at ℓ = 0.4 to 6.2% at ℓ = 0.8. For ℓ = 0.2, we see an average decrease in accuracy of 1.0% at 100 samples. In the last row of Figure 5, we see the average accuracies for the three classifiers at 100 samples, reflecting the improvements in accuracy obtained by the Gaussian process classifiers already described. We see that all classifiers tend to decrease their accuracy as the length scale decreases, which coincides with the increased complexity of classification boundaries for lower length scales seen in Figure 4. This detriment in performance becomes more pronounced between ℓ = 0.4 and ℓ = 0.2. This change corresponds with the average geodesic distance between points in the fixed design data-set, which is equal to 0.39. This metric is shown as a dashed vertical line bottom row plot of Figure 5. Classification regions with a characteristic size smaller than this value could be ignored by the classifiers, which is what we observe in the top row of Figure 5. In these cases, the uncertainty estimates used for active learning might be inadequate, leading to a worse performance compared to the longer length scale cases. Overall, we see that Gaussian process classifiers and active learning provide advantages in accuracy when compared to the baseline nearest neighbor classifier.

### 3.2. Characterization of Inducibility Regions

**Figure 6:**
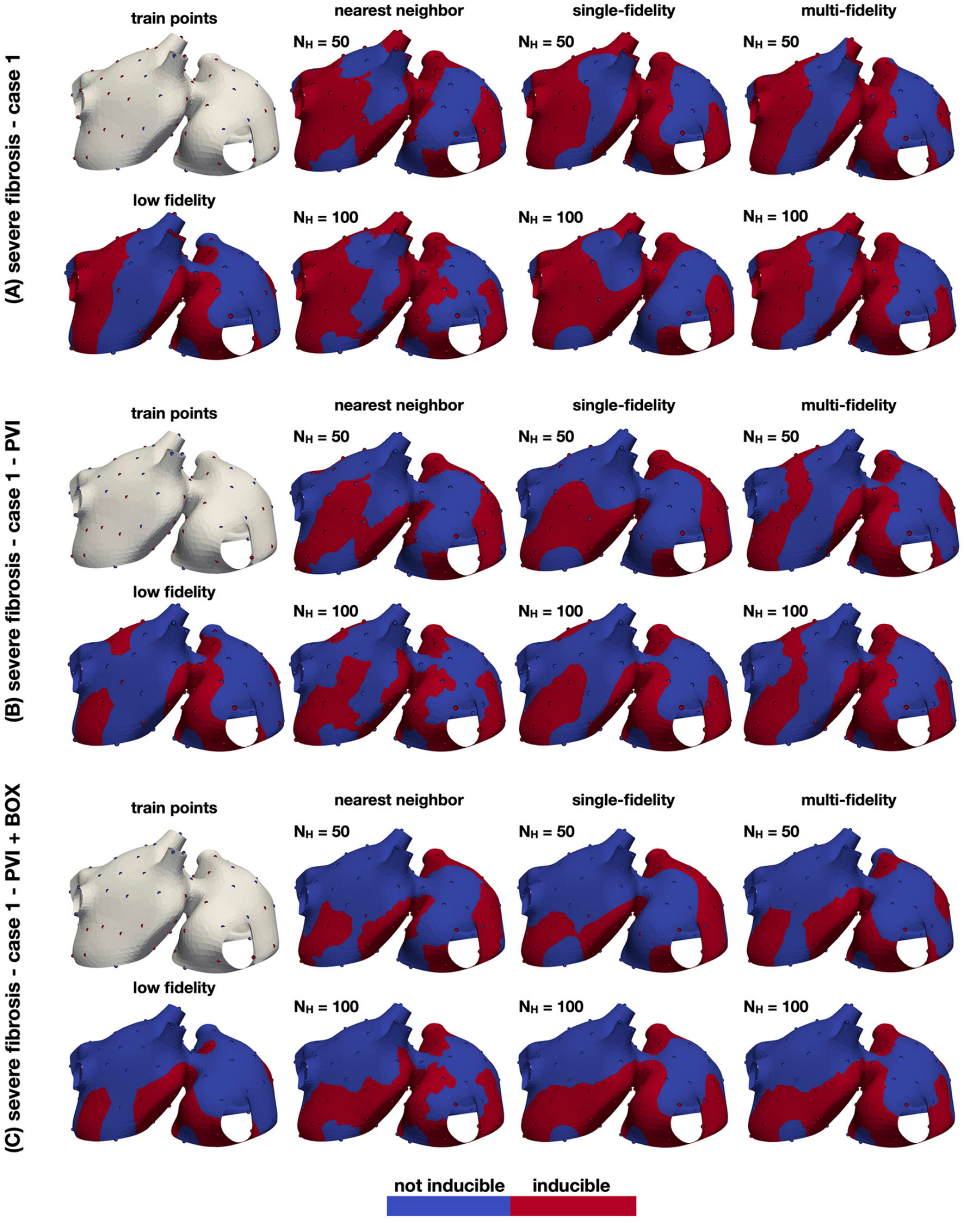
Inducibility maps for the three cases. The performance of the classifiers is analyzed for three cases: a case with no ablation **(A)**, a case with PVI ablation **(B)** and a case with PVI+BOX ablation **(C)**. In each panel, the leftmost column shows the training set (top) and the single-fidelity Gaussian process classifier trained with 100 low fidelity samples (bottom). In the remainder panels, we show the nearest neighbor, single-fidelity Gaussian process classifier, and multi-fidelity Gaussian process classifier trained with 50 and 100 high fidelity samples. The ground truth points are also shown in these panels.

We examine the inducibility of the 9 models described in Section 2.1, specifically 3 different fibrotic patterns and 3 ablation strategies: no ablation, PVI, and PVI+BOX. For each model, we create a training set and test set, both containing 100 samples, using a fixed design, as described in Section 3.1 and shown in Figure 6. We run the model using each of these points as a pacing site and check whether AF was induced or not. For the training set, we also run the low fidelity model, obtaining 100 samples. In total we run 1800 high fidelity simulations and 900 low fidelity simulations. We test three different classifiers for both cases: a nearest neighbor classifier described in Section 3.1, a single-fidelity Gaussian process classifier described in Section 2.3, and a multi-fidelity Gaussian process classifier described in Section 2.6 with 100 low fidelity samples. We train the classifiers with different amounts of data from the training set, ranging from 20 to 100 points. For each level of data, we evaluate the performance of the classifier computing the balanced accuracy in the 100 samples of the test set.

**Figure 7:**
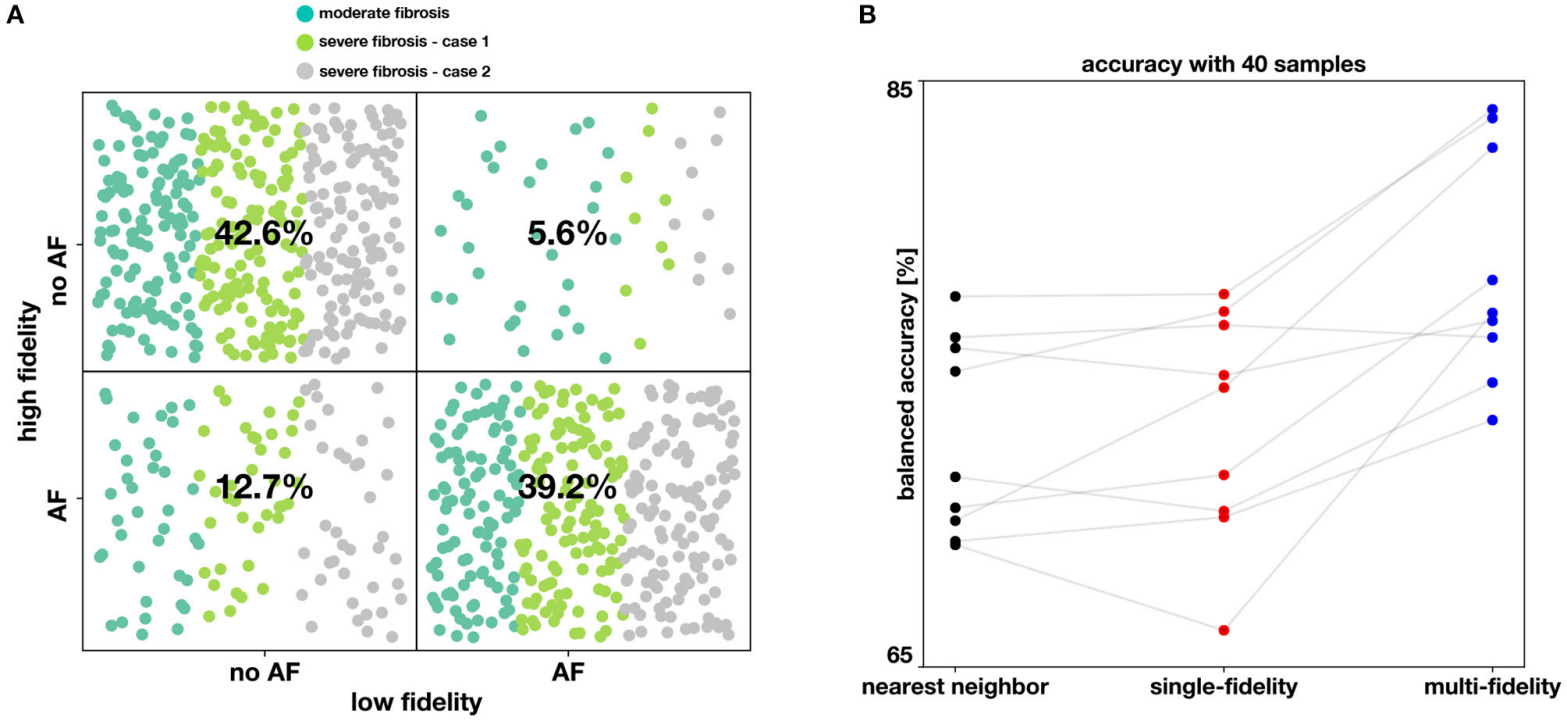
Performance of the classifiers. In **(A)**, the agreement between the low fidelity and the high fidelity model is reported as a confusion matrix, as resulting from 1,800 simulations (900 per fidelity). Moreover, each point is colored according to the case of fibrosis. In **(B)**, we compare the balanced accuracy for the nearest neighbor, single-fidelity, and multi-fidelity classifier, for all nine model scenarios and with a fixed budget of 40 high fidelity simulations.

**Figure 8:**
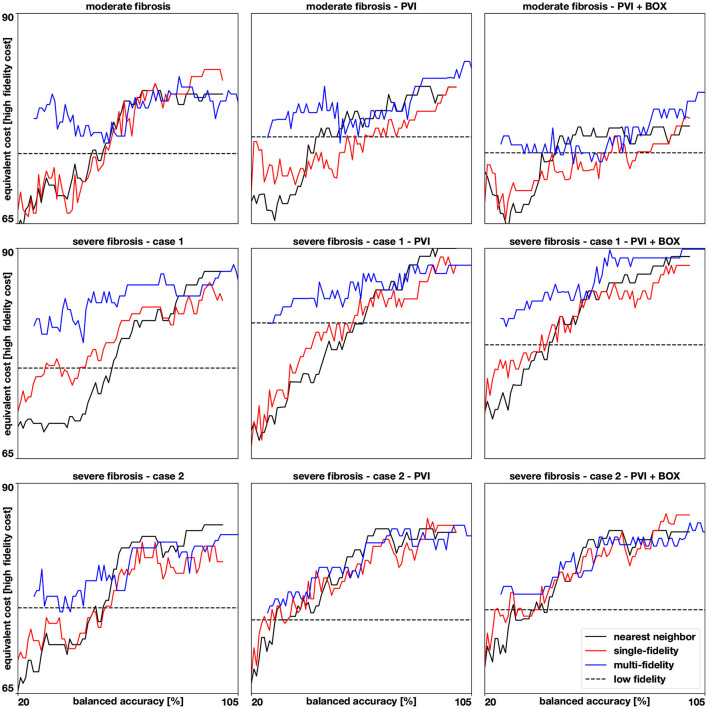
Accuracies for 9 different cases. We show how the balanced accuracy evolves as more samples (from 20 to 100) are available for the multi-fidelity, single-fidelity, and nearest neighbor classifiers. The samples are represented as the cost of running a high fidelity model and the multi-fidelity curve is shifted to the right to account for the cost of 100 low fidelity simulations. The dashed horizontal line represents the accuracy of a Gaussian process classifier trained with 100 low fidelity simulations predicting the high fidelity test set.

The results of this numerical experiment are summarized in Figures 6–8 and Table 1. First, we note that training and predicting with the Gaussian process classifiers only takes a negligible fraction of the cost of high fidelity model, less than 5 min on a laptop. In Figure 6, we show the resulting classifiers trained with the same 50 and 100 high fidelity samples and also the low fidelity classifier trained with 100 samples. We see that the multi-fidelity classifier at 50 and 100 samples shares some features with the low fidelity classifier that are not present in the other two classifiers. Nonetheless, the multi-fidelity classifier is learning from the high fidelity data, as its balanced accuracy increases as the number of samples increases, as seen in Figure 8. We observe that the differences in accuracy tend to collapse as more data is available, showing small differences when 100 samples are provided to the classifiers. The multi-fidelity classifier has the biggest advantage in the small data regime, when it is trained with between 20 and 70 high fidelity samples. Perhaps surprisingly, we see that the low fidelity classifier is always more accurate than the single-fidelity classifiers trained with 20 samples. The cost of training the low fidelity classifier is approximately equivalent to the cost of acquiring 6.25 high fidelity samples, which makes it a cost-effective alternative to estimate the inducibility with limited budget. Along the same line, we compare the accuracies of the different classifiers for the different cases when the training with the equivalent cost of 40 high fidelity simulations in Figure 7B. This is the number of simulations that has been used in clinical studies to optimize the ablation treatment (Boyle et al., 2019). We observe that by using the multi-fidelity classifier we gain, on average, 5.4% points of accuracy comparing to the single-fidelity classifier and 5.7% comparing to the nearest neighbor classifier. Only in one case there was a decrease in accuracy when using the multi-fidelity classifier, but of only 0.45% points of accuracy.

**Table 1:** Inducibility results from the 1,800 high fidelity simulations and 900 low fidelity simulations.

		**Inducibility [%]**			
**Fibrosis**	**Ablation**	**Test**	**Train**	**Low fidelity**	**Low and high fidelity agreement [%]**
Moderate	-	60	58	55	77
Moderate	PVI	52	43	41	76
Moderate	PVI+BOX	47	40	38	78
Severe - case 1	-	62	62	52	82
Severe - case 1	PVI	51	50	42	86
Severe - case 1	PVI+BOX	48	47	36	85
Severe - case 2	-	73	65	57	84
Severe - case 2	PVI	60	54	44	84
Severe - case 2	PVI+BOX	54	48	38	84

*We show the fraction of simulation where AF was inducible for 3 different fibrotic patterns and for a baseline case and 2 ablation strategies. We also report the fraction of simulations where the low and high fidelity models predicted the same outcome*.

We analyze the agreement between the low and high fidelity models by looking at training sets for all cases in Figure 7A. Overall, we find the low and high fidelity agree in 81.7% of the simulation. However, we see that the low fidelity model is biased towards predicting no AF when the high fidelity model is predicting AF. This is confirmed in every case, as can be seen in Table 1, where low fidelity inducibility is always lower than the high fidelity inducibility. A possible explanation is that the low fidelity model, being based on a coarser discretization of the atrial model, has fewer fine-grained features (fibrosis, anatomy, wall thickness) that might favor AF. It is also worth noting that we adapted the conduction velocity in the low fidelity model by increasing it to the level of the high fidelity one, a change that is potentially antiarrhythmic but that increased the correlation between the models and hence the overall performance of the multi-fidelity classifier. We also found that in the case of 50% fibrosis, the low fidelity model tends to predict proportionally more occurrence of AF when the high fidelity model is not predicting AF.

Finally, we see in Table 1 that the ablation strategies applied are decreasing the inducibility in all cases, both for the train, test and low fidelity sets. We see that pulmonary vein isolation has more impact on the inducibility than the subsequent box ablation for all cases, both in the train and the test set.

## 4. Discussion

In this study, we propose a novel methodology to estimate the AF inducibility regions of a computational model of the human atria. This is achieved by training a Gaussian process classifier that indicates whether a given point on the atria is associated with a sustained AF event, when incrementally pacing from its location. Our classifier is directly trained on the atrial surface, hence it embodies the geometrical and topological properties of the atria, which are known to be key determinants in AF. Gaussian process regression on Riemannian manifolds is not a novel concept, as well as its link to certain types of SPDEs (Lindgren et al., 2011). To the best of our knowledge, however, this is the first study proposing a multi-fidelity Gaussian process classifier on manifolds, which extends our previous work on Euclidean spaces (Sahli Costabal et al., 2019). The proposed method is non-intrusive, in the sense that the atrial model is a black-box, with comparable training cost to a nearest neighbor classifier. Moreover, when a low fidelity model is available—in our case, obtained by coarsening the computational mesh—, the accuracy of the classifier can be sensibly improved with a multi-fidelity approach. Finally, given its structure, the methodology can be easily extended to multi-class classifier, e.g., with the capability to distinguish AF episodes from atrial flutter.

From a methodological perspective, our results show that the accuracy of the classifier depends on the length scale of the inducibility region. Intuitively, the shorter the length scale is, the more training data is needed. When the length scale is much smaller than the size of the atria, it is more likely to observe an inducibility region composed of disconnected and relatively small components. Moreover, the boundary of the inducibility region becomes less smooth. Interestingly, the length scale has, however, a limited effect on the estimate of the overall inducibility. This is due to fact that the volume of the inducibility region is only marginally affected by the smoothness of its boundary and the presence of multiple disconnected regions. We attempt to estimate the length scale of the inducibility map by training a single-fidelity classifier with both the high fidelity test and train sets. The average length scale of the resulting classifier of the baseline AF model is ℓ= 0.28. This is smaller than the average distance between points in the training set, which corresponds to 0.39, and may explain the balanced accuracies that we obtained were only around 90%. We also observed in the numerical assessment that the efficiency of active learning deteriorates at smaller length scales, for ℓ between 0.2 to 0.4, and we decided not to use it for predicting inducibility maps in the experiments in Section 3.2, also to limit the computational cost.

From a computational viewpoint, the proposed multi-fidelity classifier reports the maximum improvements in accuracy in a typical data set of 40 pacing sites. In general, the multi-fidelity classifier was more accurate for a small number of samples (less than 50), while for a larger sample size the difference between single- and multi-fidelity classifiers is less pronounced. When comparing the model without ablation lines and with ablation, both high- and low fidelity models agree on the observed reduced inducibility due to ablation. In the case of ablation, therefore, it is convenient to adopt a multi-fidelity approach or even just the low fidelity classifier, to save computational time. In fact, the biggest advantage of the low fidelity classifier relies on its very limited computational cost, which is only a small fraction of the high fidelity counterpart. This highlights the importance of taking advantage of these inexpensive approximations of the high fidelity model whenever possible. We remark that our low fidelity model does not require a training phase itself, thus there is no additional offline cost.

Finally, from a modeling perspective, our results on the inducibility of AF are in agreement with those reported in the literature. Firstly, points in the proximity of fibrotic regions are more likely to induce AF (Kawai et al., 2019). Visually, there is a spatial correlation between the inducibility region (see Figure 6) and the fibrosis distribution (Figure 2). The local inducibility property may therefore reflect the local tissue properties (Boyle et al., 2021). Nonetheless, inducibility may also depend on other factors, such as an abrupt change in the fiber direction, heterogeneity in the ionic parameters, and the presence of anatomical defects or a scar. Hence, pacing sites leading to AF may not necessarily be correlated with the local tissue properties. Secondly, our results show that, with a fixed design, 40 pacing points are sufficient to achieve a good estimate of the inducibility (Boyle et al., 2021), while 20 are probably too few. The multi-fidelity classifier, however, can achieve high accuracy with only 20 samples. Thirdly, the ablation treatment reduced the overall inducibility, essentially because a large inducible region surrounding the pulmonary veins has been isolated from the rest of the tissue, impeding the emergence of AF. Due to the presence of severe fibrosis in the tissue, however, it is still possible to induce AF from several other portions of the atria, mostly unaffected by ablation. Finally, as described above, the inducibility region in both cases shows a small length scale, which can explain why pacing from different but sufficiently close points may lead to discordant results in AF inducibility. In other words, the uncertainty in the outcome is potentially large for some pacing sites.

Our work also presents some limitations. We limited our analysis to a single anatomy, but we tested different fibrosis patterns, in terms of distribution and severity, and two standard-of-care ablation strategies. Therefore, the framework can be applied with no changes to other anatomies and therapies, such as antiarrhythmic drug therapy (Sahli Costabal et al., 2018; Gharaviri et al., 2021a). It is worth to mention that for this study we ran 1 800 high fidelity simulations and 900 low fidelity ones, for a total cost of roughly 25 000 node-hours on the CSCS supercomputer. We also tested a single pacing protocol with a fixed design. The stimulation protocol is typically tailored to the ionic model and can be tested in a single-cell preparation, but sometimes this is not optimal, especially in the presence of heterogeneity and fibrosis. Optimized protocols (Azzolin et al., 2021) can be easily combined with our approach, since the algorithm does not depend on it. The duration of each simulation, 4 s, is sufficiently long to detect AF events, but it might preclude the discovery of self-terminating episodes of AF, or the translation of an AF event to atrial flutter. These cases are typically very limited in number. The presence of self-terminating AF also depends on the ionic model used, which may not be suitable for long simulations (more than 1 min). Finally, we observed that using active learning can be effective in judiciously selecting new observation sites, albeit with a deteriorating efficiency at smaller length scales. Nonetheless, this limitation motivates future work on exploring new kernel functions and active learning criteria that might be better suited for this task.

From a clinical perspective, there is an increasing application of patient-specific electrophysiology models. Thus, there is a compelling need for reducing the overall time needed to deliver the optimal virtual treatment within the constraints dictated by clinical practice (Azzolin et al., 2021; Boyle et al., 2021; Pagani and Manzoni, 2021). This study shows that the proposed Gaussian process classifier can, in fact, reduce the computational cost while maintaining a comparable or even better accuracy to a single-fidelity approach. Moreover, it does not require intrusive changes to existing implementations and it has a very limited computational overhead, rendering its translation to existing patient-specific solutions feasible and appealing.

Inducibility maps can also offer a novel, yet unexplored, view into AF, possibly unveiling regions susceptible to trigger AF. They could be used to design and test ablation scenarios, e.g., by isolating vulnerable regions. These maps could also be used to validate an AF model, by checking whether the patient-specific model and the real atria agree on the inducibility observed during a procedure.

In summary, our multi-fidelity classifier provides an efficient methodology to evaluate the effect of ablation therapy in patient-specific models of AF. We envision that this tool will accelerate the personalization of accurate treatments in the clinical setting.

## Data Availability Statement

The datasets presented in this study can be found in online repositories. The names of the repository/repositories and accession number(s) can be found at: https://github.com/fsahli/AtrialMFclass.

## Author Contributions

FS and SP: conceived the problem. FS and PP: formulated and implemented the classifier, whereas LG: implemented all necessary steps to perform multi-fidelity simulations on the supercomputer. AG and SP: provided the atrial model, the fibrosis pattern, and the ablation lines. RK and SP: supervised the work of LG. FS, SP, and LG: wrote the manuscript, and all authors reviewed and improved it. All authors contributed to the article and approved the submitted version.

## Funding

This work was supported by the ANID Millennium Science Initiative Program Grant NCN17-129, ANID-FONDECYT Postdoctoral Fellowship 3190355 to FS, the DOE grant DE-SC0019116, AFOSR grant FA9550-20-1-0060, the DOEARPA grant DE-AR0001201 awarded to PP, the Leading House for Latin American Region grant RPG 2117 to SP and FS, the Swiss Heart Foundation grant FF20042, CSCS-Swiss National Supercomputing Centre grant s1074 awarded to SP, and the SNSF grant 197041 to RK. This work was also financially supported by the Theo Rossi di Montelera Foundation, the Metis Foundation Sergio Mantegazza, the Fidinam Foundation, and the Horten Foundation.

## Conflict of Interest

The authors declare that the research was conducted in the absence of any commercial or financial relationships that could be construed as a potential conflict of interest.

## Publisher's Note

All claims expressed in this article are solely those of the authors and do not necessarily represent those of their affiliated organizations, or those of the publisher, the editors and the reviewers. Any product that may be evaluated in this article, or claim that may be made by its manufacturer, is not guaranteed or endorsed by the publisher.

## References

[B1] Azzolin L. Schuler S. Dössel O. Loewe A. (2021). A reproducible protocol to assess arrhythmia vulnerability *in silico*: Pacing at the end of the effective refractory period. Front. Physiol. 12, 420. 10.3389/fphys.2021.656411 33868025 PMC8047415

[B2] Borovitskiy V. Terenin A. Mostowsky P. Deisenroth M. (2020). Matérn gaussian processes on riemannian manifolds, in Advances in Neural Information Processing Systems, Vol. 33, eds Larochelle H. Ranzato M. Hadsell R. Balcan M. F. Lin H. (Curran Associates, Inc.), 12426–12437.

[B3] Boyle P. M. Ochs A. R. Ali R. L. Paliwal N. Trayanova N. A. (2021). Characterizing the arrhythmogenic substrate in personalized models of atrial fibrillation: sensitivity to mesh resolution and pacing protocol in af models. EP Europace 23, i3–i11. 10.1093/europace/euaa385 33751074 PMC7943367

[B4] Boyle P. M. Zghaib T. Zahid S. Ali R. L. Deng D. Franceschi W. H.. (2019). Computationally guided personalized targeted ablation of persistent atrial fibrillation. Nat. Biomed. Eng. 3, 870–879. 10.1038/s41551-019-0437-9 31427780 PMC6842421

[B5] Chen S.-A. Hsieh M.-H. Tai C.-T. Tsai C.-F. Prakash V. S. Yu W.-C.. (1999). Initiation of atrial fibrillation by ectopic beats originating from the pulmonary veins. Circulation 100, 1879–1886. 10545432 10.1161/01.cir.100.18.1879

[B6] Cohn D. A. Ghahramani Z. Jordan M. I. (1996). Active learning with statistical models. J. Artif. Intell. Res. 4, 129–145.

[B7] Colli Franzone P. Pavarino L. F. Scacchi S. (2014). Mathematical Cardiac Electrophysiology, Vol. 13. Cham: Springer.

[B8] Courtemanche M. Ramirez R. J. Nattel S. (1998). Ionic mechanisms underlying human atrial action potential properties: insights from a mathematical model. Am. J. Physiol. Heart Circ. Physiol. 275, H301–H321. 9688927 10.1152/ajpheart.1998.275.1.H301

[B9] Coveney S. Corrado C. Roney C. Wilkinson R. Oakley J. Lindgren F.. (2019). Probabilistic interpolation of uncertain local activation times on human atrial manifolds. IEEE Trans. Biomed. Eng. 67, 99–109. 10.1109/TBME.2019.2908486 30969911

[B10] Coveney S. Corrado C. Roney C. H. O'Hare D. Williams S. E. O'Neill M.D.. (2020). Gaussian process manifold interpolation for probabilistic atrial activation maps and uncertain conduction velocity. Philosoph. Trans. R. Soc. A Math. Phys. Eng. Sci. 378:20190345. 10.1098/rsta.2019.0345 32448072 PMC7287339

[B11] Crane K. Weischedel C. Wardetzky M. (2013). Geodesics in heat: a new approach to computing distance based on heat flow. ACM Trans. Graph. 32, 10. 10.1145/2516971.2516977

[B12] Dhamala J. Bajracharya P. Arevalo H. J. Sapp J. L. Horácek B. M. Wu K. C.. (2020). Embedding high-dimensional bayesian optimization via generative modeling: parameter personalization of cardiac electrophysiological models. Med. Image Anal. 62:101670. 10.1016/j.media.2020.101670 32171168 PMC7237332

[B13] Fresca S. Manzoni A. Dedè L. Quarteroni A. (2020). Deep learning-based reduced order models in cardiac electrophysiology. PLoS ONE 15:e0239416. 10.1371/journal.pone.0239416 PMC7529269 33002014

[B14] Fu Z. Kirby R. M. Whitaker R. T. (2013). A fast iterative method for solving the eikonal equation on tetrahedral domains. SIAM J. Sci. Comput. 35, C473–C494. 10.1137/120881956 25221418 PMC4162315

[B15] Gharaviri A. Bidar E. Potse M. Zeemering S. Verheule S. Pezzuto S. Krause R. Maessen J. G. Auricchio A. Schotten U. (2020). Epicardial fibrosis explains increased endo-epicardial dissociation and epicardial breakthroughs in human atrial fibrillation. Front. Physiol. 11:68. 10.3389/fphys.2020.00068 32153419 PMC7047215

[B16] Gharaviri A. Pezzuto S. Potse M. Conte G. Zeemering S. Sobota V. Verheule S. Krause R. Auricchio A. Schotten U. (2021a). Synergistic antiarrhythmic effect of inward rectifier current inhibition and pulmonary vein isolation in a 3d computer model for atrial fibrillation. EP Europace 23, i161–i168. 10.1093/europace/euaa413 33751085

[B17] Gharaviri A. Pezzuto S. Potse M. Verheule S. Conte G. Krause R. Schotten U. Auricchio A. (2021b). Left atrial appendage electrical isolation reduces atrial fibrillation recurrences: simulation study. Circ. Arrhythmia Electrophysiol. 14:e009230. 10.1161/CIRCEP.120.009230 33356357

[B18] Gneiting T. (2013). Strictly and non-strictly positive definite functions on spheres. Bernoulli 19, 1327–1349.

[B19] Gramacy R. B. Polson N. G. (2017). Particle learning of gaussian process models for sequential design and optimization. J. Comput. Graph. Stat. 20, 102–118. 10.1198/jcgs.2010.09171

[B20] Haissaguerre M. Jaïs P. Shah D. C. Takahashi A. Hocini M. Quiniou G.. (1998). Spontaneous initiation of atrial fibrillation by ectopic beats originating in the pulmonary veins. New England J. Med. 339, 659–666. 9725923 10.1056/NEJM199809033391003

[B21] Hoffman M. D. Gelman A. (2014). The no-u-turn sampler: adaptively setting path lengths in hamiltonian monte carlo. J. Mach. Learn. Res. 15, 1593–1623.

[B22] Kaboudian A. Cherry E. M. Fenton F. H. (2019). Real-time interactive simulations of large-scale systems on personal computers and cell phones: toward patient-specific heart modeling and other applications. Sci. Adv. 5:eaav6019. 10.1126/sciadv.aav6019 30944861 PMC6436932

[B23] Kapoor A. Grauman K. Urtasun R. Darrell T. (2007). Active learning with gaussian processes for object categorization, in 2007 IEEE 11th International Conference on Computer Vision (Rio de Janeiro: IEEE), 1–8.

[B24] Kawai S. Mukai Y. Inoue S. Yakabe D. Nagaok K. Sakamoto K.. (2019). Non-pulmonary vein triggers of atrial fibrillation are likely to arise from low-voltage areas in the left atrium. Sci. Rep. 9:12271. 10.1038/s41598-019-48669-1 31439861 PMC6706423

[B25] Kennedy M. C. O'Hagan A. (2000). Predicting the output from a complex computer code when fast approximations are available. Biometrika 87, 1–13. 10.1093/BIOMET/87.1.1

[B26] Krause D. Potse M. Dickopf T. Krause R. Auricchio A. Prinzen F. (2012). Hybrid parallelization of a large-scale heart model, in Facing the Multicore-Challenge II (Berlin: Springer), 120–132.

[B27] Lindgren F. Rue H. Lindström J. (2011). An explicit link between gaussian fields and gaussian markov random fields: the stochastic partial differential equation approach. J. R. Stat. Soc. Series B (Stat. Methodol.) 73, 423–498. 10.1111/j.1467-9868.2011.00777.x

[B28] Loewe A. Poremba E. Oesterlein T. Luik A. Schmitt C. Seemann G. Dössel O. (2019). Patient-specific identification of atrial flutter vulnerability–a computational approach to reveal latent reentry pathways. Front. Physiol. 9, 1910. 10.3389/fphys.2018.01910 30692934 PMC6339942

[B29] McDowell K. S. Zahid S. Vadakkumpadan F. Blauer J. MacLeod R. S. Trayanova N. A. (2015). Virtual electrophysiological study of atrial fibrillation in fibrotic remodeling. PLoS ONE 10:e0117110. 10.1371/journal.pone.0117110 25692857 PMC4333565

[B30] Neal R. (1999). Regression and classification using gaussian process priors (with discussion). Bayesian Stat. 6, 475–501.

[B31] Nickisch H. Rasmussen C. E. (2008). Approximations for binary Gaussian process classification. Mach. Learn. Res. 9, 2035–2078.

[B32] Pagani S. Manzoni A. (2021). Enabling forward uncertainty quantification and sensitivity analysis in cardiac electrophysiology by reduced order modeling and machine learning. Int. J. Numer. Methods Biomed. Eng. e3450. 33599106 10.1002/cnm.3450 PMC8244126

[B33] Perdikaris P. Venturi D. Karniadakis G. E. (2016). Multifidelity information fusion algorithms for high-dimensional systems and massive data sets. SIAM J. Sci. Comput. 38, B521–B538. 10.1137/15M1055164

[B34] Pezzuto S. Hake J. Sundnes J. (2016). Space-discretization error analysis and stabilization schemes for conduction velocity in cardiac electrophysiology. Int. J. Numer. Methods Biomed. Eng. 32:e02762. 10.1002/cnm.3450 26685879

[B35] Pezzuto S. Quaglino A. Potse M. (2019). On sampling spatially-correlated random fields for complex geometries, in International Conference on Functional Imaging and Modeling of the Heart (Bordeaux: Springer), 103–111.

[B36] Phan D. Pradhan N. Jankowiak M. (2019). Composable effects for flexible and accelerated probabilistic programming in numpyro. arXiv preprint arXiv:1912.11554.

[B37] Potse M. (2019). Inducibility of atrial fibrillation depends chaotically on ionic model parameters, in Computing in Cardiology (CinC) (Singapore), 1–4.

[B38] Potse M. Dubé B. Richer J. Vinet A. Gulrajani R. M. (2006). A comparison of monodomain and bidomain reaction-diffusion models for action potential propagation in the human heart. IEEE Trans. Biomed. Eng. 53, 2425–2435. 10.1109/TBME.2006.880875 17153199

[B39] Potse M. Gharaviri A. Pezzuto S. Auricchio A. Krause R. Verheule S. Schotten U. (2018). Anatomically-induced fibrillation in a 3d model of the human atria, in 2018 Computing in Cardiology Conference (CinC), Vol. 45 (Maastricht: IEEE).

[B40] Quaglino A. Pezzuto S. Koutsourelakis P. Auricchio A. Krause R. (2018). Fast uncertainty quantification of activation sequences in patient-specific cardiac electrophysiology meeting clinical time constraints. Int. J. Numer. Methods Biomed. Eng. 34:e2985. 29577657 10.1002/cnm.2985

[B41] Quaglino A. Pezzuto S. Krause R. (2019). High-dimensional and higher-order multifidelity monte carlo estimators. J. Comput. Phys. 388, 300–315. 10.1002/cnm.2985 29577657

[B42] Rasmussen C. E. Williams C. K. I. (2006). Gaussian Processes for Machine Learning. (Cambridge: MIT Press).

[B43] Roney C. H. Whitaker J. Sim I. O'Neill L. Mukherjee R. K. Razeghi O.. (2019). A technique for measuring anisotropy in atrial conduction to estimate conduction velocity and atrial fibre direction. Comput. Biol. Med. 104, 278–290. 10.1016/j.compbiomed.2018.10.019 30415767 PMC6506689

[B44] Sahli Costabal F. Perdikaris P. Kuhl E. Hurtado D. E. (2019). Multi-fidelity classification using gaussian processes: accelerating the prediction of large-scale computational models. Comput. Methods Appl. Mech. Eng. 357:112602. 10.1016/j.cma.2019.112602

[B45] Sahli Costabal F. Seo K. Ashley E. Kuhl E. (2020). Classifying drugs by their arrhythmogenic risk using machine learning. Biophys. J. 118, 1165–1176. 10.1016/j.bpj.2020.01.012 32023435 PMC7063479

[B46] Sahli Costabal F. Yao J. Kuhl E. (2018). Predicting drug-induced arrhythmias by multiscale modeling. Int. J. Numer. Methods Biomed. Eng. 118, 1165–1176. 10.1002/cnm.2964 29424967

[B47] Verma A. Jiang C.-Y. Betts T. R. Chen J. Deisenhofer I. Mantovan R.. (2015). Approaches to catheter ablation for persistent atrial fibrillation. New Engl. J. Med. 372, 1812–1822. 10.1056/NEJMoa1408288 25946280

[B48] Virani S. S. Alonso A. Aparicio H. J. Benjamin E. J. Bittencourt M. S. Callaway C. W.. (2021). Heart disease and stroke statistics–2021 update: a report from the american heart association. Circulation 143, e254–e743. 10.1161/CIR.0000000000000950 33501848 PMC13036842

[B49] Whittle P. (1963). Stochastic-processes in several dimensions. Bull. Int. Stat. Inst. 40, 974–994.

[B50] Zaman M. S. Dhamala J. Bajracharya P. Sapp J. L. Horácek B. M. Wu K. C.. (2021). Fast posterior estimation of cardiac electrophysiological model parameters via bayesian active learning. Front. Physiol. 12:740306. 10.3389/fphys.2021.740306 34759835 PMC8573318

